# New lesion segmentation for multiple sclerosis brain images with imaging and lesion-aware augmentation

**DOI:** 10.3389/fnins.2022.1007453

**Published:** 2022-10-21

**Authors:** Berke Doga Basaran, Paul M. Matthews, Wenjia Bai

**Affiliations:** ^1^Department of Computing, Imperial College London, London, United Kingdom; ^2^Data Science Institute, Imperial College London, London, United Kingdom; ^3^Department of Brain Sciences, Imperial College London, London, United Kingdom; ^4^UK Dementia Research Institute, Imperial College London, London, United Kingdom

**Keywords:** multiple sclerosis, new lesion detection, data augmentation, nnU-Net, MRI, longitudinal lesion segmentation, biomedical segmentation

## Abstract

Multiple sclerosis (MS) is an inflammatory and demyelinating neurological disease of the central nervous system. Image-based biomarkers, such as lesions defined on magnetic resonance imaging (MRI), play an important role in MS diagnosis and patient monitoring. The detection of newly formed lesions provides crucial information for assessing disease progression and treatment outcome. Here, we propose a deep learning-based pipeline for new MS lesion detection and segmentation, which is built upon the nnU-Net framework. In addition to conventional data augmentation, we employ imaging and lesion-aware data augmentation methods, axial subsampling and CarveMix, to generate diverse samples and improve segmentation performance. The proposed pipeline is evaluated on the MICCAI 2021 MS new lesion segmentation challenge (MSSEG-2) dataset. It achieves an average Dice score of 0.510 and *F*_1_ score of 0.552 on cases with new lesions, and an average false positive lesion number *n*_*FP*_ of 0.036 and false positive lesion volume *V*_*FP*_ of 0.192 *mm*^3^ on cases with no new lesions. Our method outperforms other participating methods in the challenge and several state-of-the-art network architectures.

## 1. Introduction

Multiple sclerosis (MS) is a chronic inflammatory neurological disease affecting the central nervous system (CNS). Generally detected in young adults, ages 20–40, demyelinated lesions in the CNS lead to cognitive and physical disabilities, affecting vision, learning and memory, musculoskeletal system, and internal organ dysfunctions (Ghasemi et al., 2017). While MS is not fatal, average life expentancy is 5–10 years lower than average. The McDonald diagnostic criteria (Thompson et al., 2018) for MS provides guidelines for diagnosing the patient based on the number of lesions, lesion size, and locations of lesions in the brain and spinal cord. Disease progression for MS patients is highly varied and unpredictable, therefore, identifying disease trajectories and closely following them are important for prognosis and treatment decisions.

Multiple sclerosis is typically diagnosed *via* the patient showing symptoms in combination with supporting medical imaging of the brain. Specifically, the presence of lesions on brain MRI scans is a predictive image-based biomarker for MS diagnosis. Common multi-modal brain MRI acquisitions are composed of T1, T2, fluid-attenuated inversion recovery (FLAIR) and proton-density modalities. Lesions in the periventricular, juxtacortical, and infratentorial regions are presented as hyperintensities on T2-weighted and FLAIR MRI, or hypointensities on T1-weighted MRI (Filippi et al., 2019).

To monitor the progression of the disease, patients may take multiple MRI scans at different time points, typically 6–12 months apart. The detection of newly formed lesions provides crucial information for assessing disease activity and treatment outcome. Formation of new lesions correlates with the progression and severity of the disease and is often complemented with increased symptoms (Weiner et al., 2000). Manual assessment of these imaging scans can be time consuming, especially when attempting to identify formations of new lesions compared to the baseline scan. Automated detection and segmentation of brain lesions substantially aid neuro-radiologists in tracking the progression of the disease. Additionally, state-of-the-art machine learning methods can provide fast and reliable quantitative information on detected abnormalities, such as lesion load, lesion number, or even patient outcome (Tousignant et al., 2019; McKinley et al., 2020).

Recent developments in convolutional neural networks (CNNs) have shown promising results for image segmentation tasks (Alzubaidi et al., 2021). The two-dimensional (2D) U-Net (Ronneberger et al., 2015) and three-dimensional (3D) U-Net (Çiçek et al., 2016) architectures have been widely adopted in biomedical image segmentation tasks due to their ability in incorporating multi-scale spatial context and generalisability across different biomedical domains. nnU-Net (Isensee et al., 2021a), a U-Net based medical image segmentation network which employs a self-adapting framework, has shown excellent performance in a number of organ segmentation tasks (Isensee et al., 2021a,b). nnU-Net stands for “no new U-Net.” Its strong performance across a variety of datasets is not due to a new network architecture, but rather to automating the process of manual configuration of setting up a neural network. nnU-Net configures its network and pipeline subject to dataset properties and available GPU memory budget, maximizing the training patch size which the GPU memory will allow.

Nevertheless, there are still several challenges in applying these methods to brain image segmentation tasks, such as for MS lesion segmentation. The first challenge is the scarcity of data and annotation. Most of the public MS lesion datasets, such as the 2016 MSSEG (Commowick et al., 2018) and the 2015 ISBI MS (Carass et al., 2017) datasets, only contain images from a dozen of subjects. In a field where data diversity is paramount, data augmentation methods become critical tools to boost model performance. The second challenge is the class imbalance problem. In MS lesion segmentation, almost all of the foreground voxels represent healthy brain tissues and the lesions only constitute for a minority of the voxels. This means that the deep learning models tend to learn from the healthy tissues instead of the lesions of interest. In an attempt to allow the network to learn features from underrepresented classes, patches which contain the underrepresented class are often oversampled (Rahman and Davis, 2013). Despite oversampling strategies, the class imbalance problem is amplified even more when working with longitudinal MS data, where the objective is to detect new lesions. New lesions to detect in follow-up scans can make up as little as 0.01% of the 3D image volume.

There is still room for improvement for current lesion segmentation methods in detecting small lesions and tracking their temporal trajectories in disease progression. Commowick et al. (2018) finds that lesion detection rates fall significantly as lesion volumes decrease, resulting in false negative results in automated segmentation. This forms a critical challenge when newly formed lesions need to be considered for MS progression monitoring, which these lesions are often small and hard to detect.

In this paper, we propose a deep learning pipeline for new MS lesion segmentation. The developed pipeline is built upon the nnU-Net framework and we incorporate multiple brain-image preprocessing steps as well as imaging and lesion-aware data augmentation techniques. We evaluate the pipeline on the MSSEG-2 challenge dataset (Commowick et al., 2021a), which demonstrates promising results for both new lesion cases and no new lesion cases.

## 2. Methods

### 2.1. Related works

#### 2.1.1. Deep learning for MS lesion segmentation

There have been contributions to machine learning methods specifically for MS lesion segmentation. Numerous methods were developed following the 2015 ISBI Longitudinal Multiple Sclerosis Lesion Segmentation Challenge (Carass et al., 2017). Valverde et al. (2017) employed a cascade of two 3D patch-wise CNNs, where the first CNN proposed candidate lesion voxels and the second one reduced falsely classified voxels. Birenbaum and Greenspan (2017) developed a multi-view longitudinal CNN and utilized priors about lesion intensities and spatial distribution to extract candidate lesions. Similar to Valverde et al., and Birenbaum et al. also used 3D patches for model training. Contrary to patch-based training, Aslani et al. (2019) proposed a multi-branch CNN which takes whole slices of the brain as input. Three 2D ResNets were separately trained for the axial, sagittal, and coronnal planes, the outputs of which were fused to generate a final 3D segmentation. Zhang et al. (2019) developed a fully convolutional densely connected network (Tiramisu) using a 2.5-dimensional input where slices were stacked from three anatomical planes, providing both global and local context in segmentation.

Transformer networks are now a widely adopted network model for both natural language processing and computer vision tasks due to their self-attention mechanisms. The Vision Transformer (ViT) (Dosovitskiy et al., 2021) showcased that a pure transformer applied on sequences of image patches can achieve competitive image classification performance. Consequently, a multitude of transformer-based frameworks for medical image segmentation have been proposed. The majority of these models utilize CNNs in conjunction with transformers, taking advantage of both local and global context information extraction. TransBTS performs 3D CNN encoding followed by a transformer for global feature modeling in multi-modal brain tumor segmentation (Wang et al., 2021). TransUNeT employs a hybrid CNN-Transformer architecture for multi-organ abdominal image segmentation (Chen et al., 2021). UNETR implements a pure transformer encoder based on ViT in combination with resolution-wise convolutions and a deconvolutional layer for decoding the image back into the original dimension (Hatamizadeh et al., 2022). It performs competitively with state-of-the-art methods in multi-organ CT and MRI brain tumor segmentation tasks.

#### 2.1.2. Data augmentation

Data augmentation can be classified into four categories: affine transformations, elastic transformations, intensity alterations, and incorporation of synthetic data. Affine transformations include flipping, rotation, scaling, and shearing of the image. Affine transformations do not drastically change the shape characteristics of the abnormal region with respect to its surrounding tissue. Elastic transformations generate a displacement grid with random displacements, which is used to deform individual voxels of the input image (Çiçek et al., 2016). The non-linear transformations alter the boundaries of the abnormal region with respect to its surrounding tissue, producing diverse samples. Intensity alterations introduce Gaussian noise, Gaussian blurring, sharpening, salt and pepper noise, and gamma augmentation etc. to improve model robustness against intensity distribution shift, which concerns imaging scans acquired from different scanner models, scanner acquisition parameters, or scanner strengths. Synthetic data augmentation utilizes generative models or MixUp (Zhang et al., 2018) techniques to generate new samples. For example, generative adversarial networks (GANs) (Goodfellow et al., 2014) were introduced for data augmentation in biomedical image segmentation (Shin et al., 2018; Sandfort et al., 2019; Hong et al., 2021). MixUp (Zhang et al., 2018) and related methods such as MixMatch (Berthelot et al., 2019) and CutMix (Yun et al., 2019) designed specific operations on two or more images to generate new samples. For brain lesion images, a lesion-aware augmentation method, CarveMix (Zhang et al., 2021), was proposed to combine two brain MRI scans to increase training data diversity. CarveMix randomly extracts lesion regions on the sagittal plane from one image and overlays them onto a target image (Zhang et al., 2021).

### 2.2. Proposed pipeline

**Figure 1:**
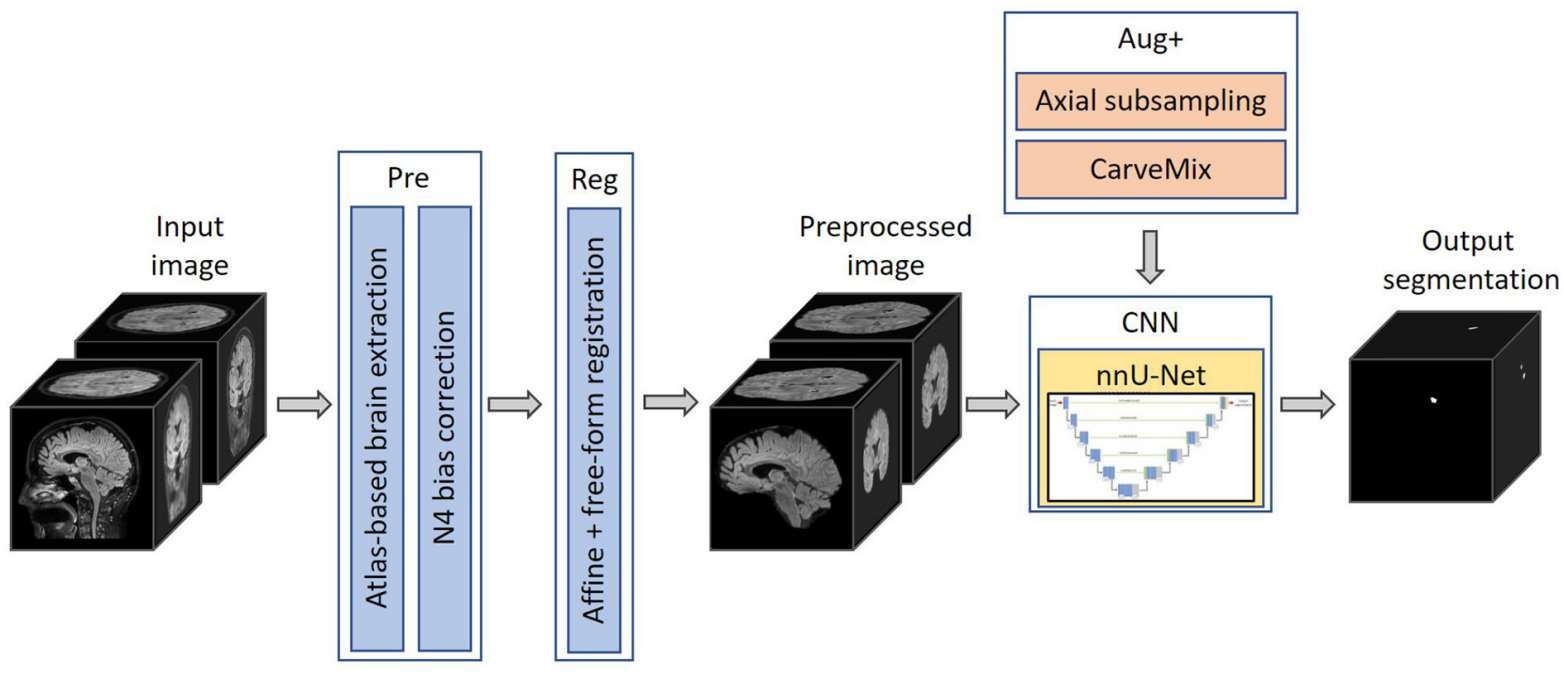
The proposed 3D framework for multiple sclerosis new lesion detection and segmentation. Framework comprises of brain extraction and N4 bias correction (Pre), intra-subject registration (Reg), imaging and lesion-aware data augmentations (Aug+), and nnU-Net. The pipeline takes input the baseline and follow-up FLAIR MRI scans, and outputs the proposed binary segmentation for new lesions.

The proposed pipeline consists of a brain image preprocessing step, followed by nnU-Net (Isensee et al., 2021a) for lesion segmentation, which is trained with imaging and lesion-aware data augmentation. An overview of the pipeline is presented in Figure 1.

#### 2.2.1. Preprocessing

Skull is stripped using an atlas-based brain extraction tool (Doshi et al., 2013) followed by N4 bias field correction (Tustison et al., 2010). This is implemented using the MSSEG-2 longitudinal preprocessing script on Anima^1^ provided by the challenge organizers. In addition, as the segmentation problem concerns imaging scans taken at different time points, we also perform intra-subject image registration so that scans of the same subject can be aligned and new lesions can be better differentiated. Since new lesions are defined on the follow-up scan, we register the baseline scan to the space of the follow-up scan. Affine image registration is performed, followed by free-form deformation, implemented using the MIRTK toolbox (MIRTK, 2021) using normalized mutual information as the loss function. Free-form deformation assists lesion segmentation in two ways: (1) brain structures, such as gyri, ventricles etc., are better registered; (2) lesions which slightly grow between scans are elastically registered so that the subsequent segmentation network can focus more on newly formed lesions.

#### 2.2.2. Segmentation network

We adopt nnU-Net (Isensee et al., 2021a) as the segmentation network, with a two-channel input: preprocessed baseline scan and preprocessed follow-up scan. The output of the network is a binary 3D prediction of new lesions which have formed in the follow-up scan. The network consists of six resolution levels, formed from contracting and expanding paths. On the contracting path, each resolution level consists of two convolutional layers, each with a 3 × 3 × 3 convolution kernel, followed by instance normalization and LeakyReLU operation. At the start of each resolution level, the first convolution has a stride of (2,2,2), which effectively downsamples the feature map. At the lowest resolution level, the first convolution has a stride of (2,1,2).

**Figure 2:**
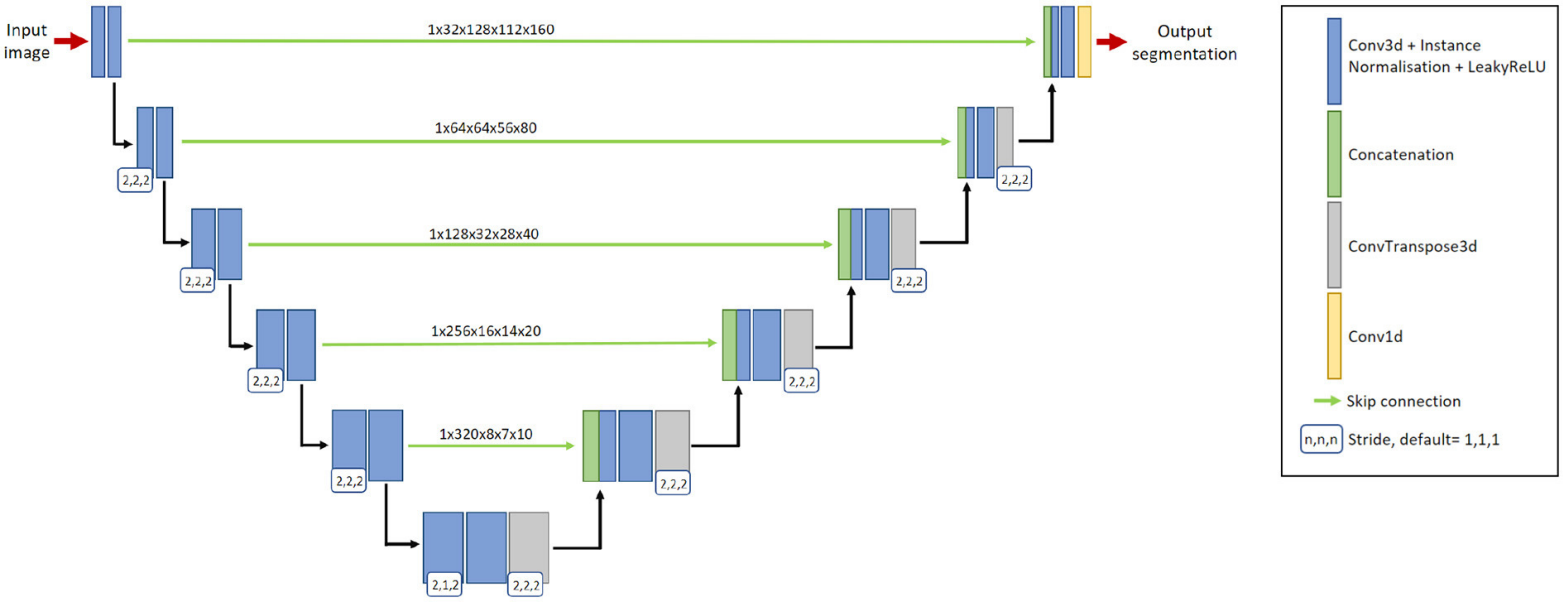
The 3D nnU-Net architecture for segmenting new lesions. It contains six resolution levels formed from contracting and expanding paths, and skip connections to recover fine-grain detail from the contracting path. The input to the network is a pair of baseline image and follow-up image. The output is the prediction of the new lesions.

On the expanding path, each resolution level consists of two convolutional layers with a 3 × 3 × 3 convolution kernel, followed by instance normalization and LeakyReLU operations, followed by an additional transposed 2 × 2 × 2 convolution operation. The transposed convolution has a stride of (2,1,2) at the lowest resolution level, and a stride of (2,2,2) at all other resolution levels. By utilizing skip connections, features extracted from the contracting path are concatenated with features at the expanding path at their respective resolution level. The network uses 32-dimensional features maps at the highest resolution layer, which is increased to 320 feature maps at the lowest resolution layer. Please refer to Figure 2 for a graphical representation of the architecture.

#### 2.2.3. Hyperparameters and implementation details

We implement the 3D full-resolution U-Net model of nnU-Net, using the 3d_fullres configuration, utilizing PyTorch. A single NVidia Tesla T4 GPU with 16GB RAM is used. Due to the GPU memory limit, 3D patches of size 128 × 112 × 160 are extracted from the original 3D images for model training. Patches are drawn randomly from the image with a 67% probability, and are ensured to include the lesion region with a 33% probability. The network is trained using a combination of Dice and cross-entropy loss, formulated as,


(1) $$\begin{array}{l} L=-\frac{2}{\left|K\right|}\underset{k\in K}{\sum }\frac{\sum_{i\in I}{ŷ}_{i\left(k\right)}y_{i\left(k\right)}}{\sum_{i\in I}{ŷ}_{i\left(k\right)}+y_{i\left(k\right)}}-\underset{i\in I}{\sum }\underset{k\in K}{\sum }y_{i\left(k\right)}\log{ŷ}_{i\left(k\right)} \end{array}$$

where *k* denotes the class, *K* denotes the number of classes (*K* = 2 in our method), *i* denotes a given voxel, *I* denotes the set of voxels over the image, ŷ is the softmax output of the segmentation network, *y* is the one-hot encoding of the ground truth label for the new lesions, and subscript *i*(*k*) is the number of voxels in the training patch for class *k*.

We use the stochastic gradient descent optimizer with Nesterov momentum of 0.99, an initial learning rate of 0.01, a polynomial learning rate decay and a batch size of 2 patches. When developing the model on the training data, five-fold cross-validation is used. Each model is trained for 1,000 epochs. After training, for each fold, we select the model which produces the highest Dice score. For inference, we ensemble segmentation outputs from the five models from each fold. No post-processing step is applied.

#### 2.2.4. Imaging and lesion-aware data augmentation

Incorporation of data augmentation methods increases model generalizability and robustness, and decreases overfitting. We utilize multiple data augmentation techniques, including the default augmentations that nnU-Net provides in the batchgenerators data augmentation framework (Isensee et al., 2020). These augmentations include mirroring, rotating, scaling, channel translation to simulate registration errors, elastic deformations, linear downsampling, brightness and contrast augmentation, gamma augmentation, Gaussian and Rician noise augmentation, and random cropping.

**Figure 3:**
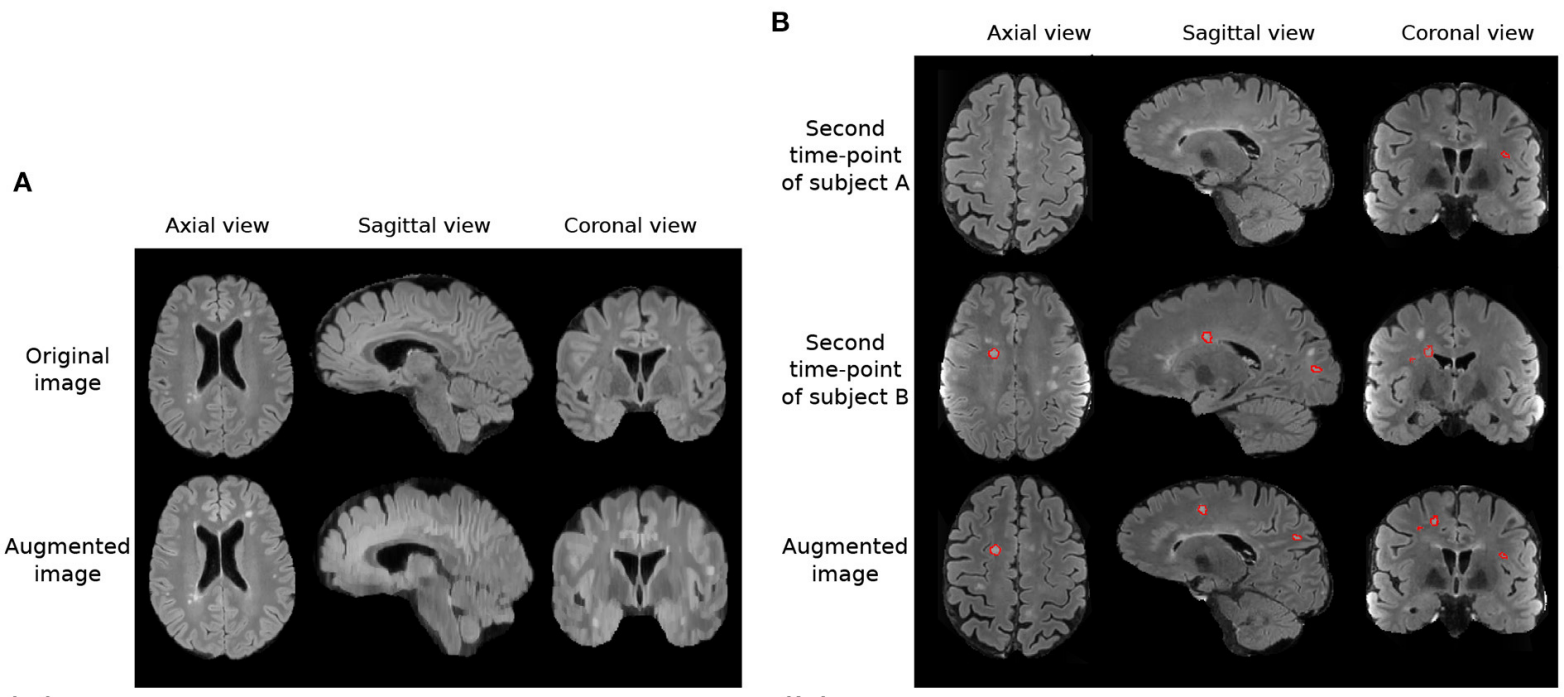
Imaging and lesion-aware data augmentations applied on the MSSEG-2 training set. **(A)** Example of axial subsampling (*n* = 4) to simulate the blurring in image acquisition. **(B)** Example of the CarveMix augmentation. Lesions from subject B are carved out and fused onto scan from subject A. Contours delineate lesion labels.

In addition to these augmentations, inspired by Kamraoui et al. (2021, 2022), we introduce axial subsampling to simulate the image acquisition process on the axial plane. Brain MRI typically acquires a stack of 2D image slices in the axial plane to form a 3D volume, which can be of a high resolution within the axial plane but subject to low resolution across the plane (Chai et al., 2020). Axial subsampling augmentation is performed by applying a median filter of size [1 × 1 × *n*] where *n* ∈ 2, 3, 4 to the axial image slices. This effectively blurs the image in the sagittal and coronal planes. Figure 3A illustrates an example of axial subsampling.

Finally, to increase the diversity of lesion images, a lesion-aware data augmentation method, CarveMix (Zhang et al., 2021), is used. CarveMix extracts a 3D region of interest (ROI) according to the lesion location and shape from one subject and mixes it with the brain image of another subject, thus creating augmented training samples. To increase diversity in augmentation, the lesion-aware ROI is generated by thresholding the distance transform of the lesion using a random threshold (Zhang et al., 2021). A synthetic image, **X**, and its label, **Y**, is generated by,


(2) $$X=X_{i}⊙M_{i}+X_{j}⊙(1-M_{i})$$

(3) $$Y=Y_{i}⊙M_{i}+Y_{j}⊙(1-M_{i})$$

where {**X**_*i*_, **Y**_*i*_} denotes one pair of image and label, {**X**_*j*_, **Y**_*j*_} denotes a second pair of image and label, **M**_*i*_ denotes the binary mask of the ROI, and ⊙ represents voxel-wise multiplication. We randomly select two subjects for CarveMix augmentation when training. Incorporation of CarveMix data augmentation increases the total volume which the lesion class covers in an image, thus reducing the effect of class imbalance caused from the foreground class making up a small percentage of the overall image. Figure 3B illustrates an example of CarveMix augmentation.

## 3. Results

### 3.1. Data

We evaluate the pipeline on the *MICCAI 2021 MS new lesion segmentation challenge* dataset (MSSEG-2) (Commowick et al., 2021a), which provides 3D FLAIR images of 100 MS patients. The images were acquired from 15 different scanners, six of them 1.5T and nine of them 3T, including three GE scanners, six Philips scanners, and six Siemens scanners. Dataset scanner information can be found at the MICCAI 2021 MSSEG-2 challenge demographics data (Commowick et al., 2022). The images have varying image size and voxel spacing, which we resample to the median spacing of the dataset, 0.977 × 0.977 × 0.530*mm*^3^, before model training. Each patient was scanned twice, with 1–3 years between the two time points, constituting for a total of 200 images. Only new lesions at the second time point were annotated. Existing lesions, growing or shrinking lesions were not delineated. Each patient was annotated by four neuroradiologist and one consensus new lesion mask was provided. We use the consensus lesion masks as ground truth for model training and evaluation.

The dataset has been partitioned into 40 training and 60 test subjects by the challenge organizers. Of the 40 training subjects, 11 of them do not exhibit new lesions, which are referred to as “no-new lesion cases.” We exclude these 11 no-new lesion cases from the training set, utilizing the remaining 29 cases for model training. Of the 60 test subjects, 28 of them do not exhibit new lesions. We use all 60 subjects for testing.

### 3.2. Evaluation metrics

The method is evaluated using the Anima analyzer tool's animaSegPerfAnalyzer^2^ function, provided by the MSSEG-2 challenge organizers in order to provide a fair comparison with other participating methods. In line with the MSSEG-2 evaluation, we use the default configuration of animaSegPerfAnalyzer, which excludes lesion volumes smaller than 3mm^3^. The performance is evaluated separately for patients with new lesions and those with no-new lesions on the test set. For the new lesion cases, we report new lesion detection and segmentation performance, true positive lesion count, false positive lesion count, and false negative lesion count; for the no-new lesion cases, we calculate the number of new lesions detected (false positive lesions) and the volume of these false positive lesions.

#### 3.2.1. Performance on new lesion cases

New lesion detection performance is evaluated using the *F*_1_ score. The *F*_1_ score measures how many lesions are correctly or incorrectly detected, regardless of the precision of its contours. It is formulated as,


$$F_{1}=2\frac{S_{L}\cdot P_{L}}{S_{L}+P_{L}}$$

where *S*_*L*_ denotes the lesion detection sensitivity (recall) and *P*_*L*_ denotes the positive predictive value (precision). The optimal *F*_1_ score is 1. A lesion is considered as being detected or true positive if the automatic detection overlaps with at least 10% of the ground truth lesion volume and does not go outside by more than 70% of the volume (Commowick et al., 2018).

New lesion segmentation performance is evaluated using the Dice similarity coefficient, DSC, which measures spatial overlap. DSC is formulated as,


$$\text{DSC}=2\frac{|A\cap G|}{|A|+|G|}$$

where *A* denotes the automatic segmentation and G denotes the ground truth. The optimal DSC is 1.

In addition to the metrics used in the MSSEG-2 challenge, we also present results for average true positive lesion count, *n*_*TP*_, average false positive lesion count, *n*_*FP*_, and average false negative lesion count, *n*_*FN*_. Average true positive lesion count evaluates the average correctly detected lesions by the automated method. *n*_*FP*_ evaluates the average incorrectly detected lesions by the automated method. Finally, *n*_*FN*_ evaluates the lesions not detected by the automated method. These metrics are averaged over the 32 new lesion cases in the test set. The consensus ground truth segmentation contains a total of 224 new lesions, therefore the optimal average true positive lesion count, *n*_*TP*_, is 7 ($\frac{224}{32}$). The optimal score for *n*_*FP*_ or *n*_*FN*_ is 0.

#### 3.2.2. Performance on no-new lesion cases

For no-new lesion cases, the number and volume of falsely predicted lesions are evaluated. To count the number of false positive lesions, the Anima tool, animaConnectedComponents^3^ function is used with default parameters. The volume of false positive lesions is calculated by multiplying the number of lesion voxels by voxel spacing. We denote number of false positive lesions as *n*_*FP*_, and volume of false positive lesions as *V*_*FP*_. The optimal scores for both false positive lesion number and volume are 0.

### 3.3. Results

#### 3.3.1. Comparison against participating methods in the challenge

The proposed pipeline is compared against MSSEG-2 participating methods and also four expert raters (Commowick et al., 2021b), reported in Table 1. The performance of MSSEG-2 participating methods and four expert raters is acquired from Commowick et al. (2021b). Table 1 shows that the proposed pipeline ranks competitively against methods submitted to the MSSEG-2 challenge. For the new lesion cases, it outperforms the other methods in terms of both the average DSC and the average *F*_1_ scores. Also, our method outperforms three of the experts in *n*_*TP*_ and *n*_*FN*_ metrics. Our method correctly identifies 24 of the 32 new lesion cases as having new lesions. We achieve comparable performance to Experts 1, 2, 3 and 4, which correctly identify 26, 25, 27, and 22 of the 32 new lesion cases as having new lesions, respectively. A non-zero *F*_1_ score is regarded as a method having correctly identified a new lesion case.

**Table 1:** Comparison of the proposed method to the challenge participating methods in terms of DSC, *F*_1_ scores, the number of true positive lesions *n*_*TP*_, the number of false positive lesions *n*_*FP*_, the number of false negative lesions *n*_*FN*_, and volume of false positive lesions *V*_*FP*_ (unit: *mm*^3^), averaged across cases.

	**New lesion cases**					**No-new lesion cases**	
	**(*n* = 32)**					**(*n* = 28)**	
**Method**	**DSC**	***F_1_***	***n_TP_***	***n_FP_***	***n_FN_***	***n_FP_***	***V_FP_***
*Expert 1*	0.629	0.709	6.063	1.281	1.094	0.036	1.453
*Expert 3*	0.597	0.637	4.500	0.844	2.375	0.000	0.000
*Expert 2*	0.535	0.601	4.313	1.094	2.500	0.107	3.981
*Expert 4*	0.459	0.519	4.469	0.594	2.375	0.036	0.623
**Proposed**	**0.510**	**0.552**	4.969	2.031	2.281	**0.036**	**0.192**
MedICL	0.507	0.500	5.344	5.063^††^	1.875	0.536^†*††*^	12.713
LaBRI-IQDA	0.500	0.515	5.563	6.094^††^	1.656	1.143^††^	38.486^*^
SNAC	0.485	0.514	5.219	3.689^†^	2.031	0.321	5.726
LaBRI-D&E	0.472	0.496	5.500	9.156^†*††*^	1.750	1.964^††^	177.131
NVAUTO	0.469	0.464^*^	5.344	12.000^†*††*^	1.906	3.286^†*††*^	68.211^*^
LaBRI-Iw	0.453^*^	0.463^*^	5.000	6.719^††^	2.250	0.857^†^	27.761^*^
New Brain	0.451^***^	0.476^***^	4.032	2.903	3.355	0.786^†^	12.371
ITU	0.443	0.480	4.688	3.094	2.438	0.148	1.487
Mediaire-B	0.437^**^	0.541	**5.688**	4.469^††^	**1.500**	0.536^†*††*^	29.235^*^
Mediaire-A	0.432^***^	0.524	5.156	3.500	2.031	0.429^†^	15.908^*^
Empenn	0.424^*^	0.532	4.178	2.719	3.031	0.286^††^	4.258^*^
McEwan-IM	0.423^***^	0.453^*^	5.469	8.531^†*††*^	1.781	0.857^†^	16.504
PVG	0.414^***^	0.449^*^	4.032	2.903	3.355	0.107	1.031
Neuropoly-1	0.411^***^	0.425^***^	3.625	2.813	3.563	0.286^†^	8.615
IAMLAB	0.411^***^	0.412^***^	5.094	6.844^†*††*^	2.156	1.679^†*††*^	19.753^*^
LYLE	0.409^***^	0.443^**^	3.406	**1.250**	3.594	**0.036**	0.470
Neuropoly-2	0.409^***^	0.413^***^	3.656	1.906	3.469	0.107	0.498
SCAN	0.403^***^	0.431^**^	4.156	2.406	3.031	0.071	5.373
SCA-SimpleUNet	0.400^***^	0.448^*^	5.406	6.344^†*††*^	1.813	0.750^†*††*^	31.232^*^
I3M	0.398^***^	0.358^***^	4.250	4.313^†^	3.000	0.393	14.800
Neuropoly-3	0.379^***^	0.416^***^	3.719	2.625	3.500	0.321^†^	19.240
The NoCoDers	0.365^***^	0.381^***^	4.750	7.594^†*††*^	2.500	1.370^†*††*^	25.848^*^
Vicorob	0.357^***^	0.369^***^	3.906	4.094^††^	3.156	0.964^†^	88.402
HufsAIM	0.346^***^	0.407^***^	2.938	1.979	4.156	0.444^†^	17.128^*^
CMIC	0.330^***^	0.362^***^	3.906	6.094^†*††*^	3.344	4.714^†*††*^	123.442
MIAL	0.309^***^	0.332^***^	4.516	6.097^†^	2.774	1.464^††^	177.861
SCA-withPriors	0.223^***^	0.216^***^	2.750	6.719^††^	4.219	2.464^†*††*^	302.121
LIT	0.214^***^	0.242^***^	2.406	11.063	4.469	0.607^†^	35.404
IBBM	0.155^***^	0.145^***^	1.906^††^	7.625^†*††*^	5.188^†^	3.786^†*††*^	123.309^***^
Optimal score	1.000	1.000	7.000	0.000	0.000	0.000	0.000

The methods are sorted in the descending order of DSC. Best results are in bold. Asterisks indicate statistical significance (^*^p ≤ 0.05, ^**^p ≤ 0.01, and ^***^p ≤ 0.005) when using a paired Student's *t*-test compared to the proposed method. We implement the Mann-Whitney *U*-test for *n*_*TP*_, *n*_*FP*_, and *n*_*FN*_ metrics due to their non-normal distribution. The dagger symbols indicate statistical significance (^†^*p* ≤ 0.05, ^††^*p* ≤ 0.01, and ^†*††*^*p* ≤ 0.005) when using a Mann-Whitney *U*-test compared to the proposed method.

For the no-new lesion cases, the proposed pipeline achieves the lowest metrics for false positive lesions, including the average number *n*_*FP*_ and the average volume *V*_*FP*_. It correctly identifies 27 out of 28 no-new lesion cases as subjects with no-new lesions. When comparing to expert raters, on the new lesion cases, the proposed pipeline outperforms Expert 4 in terms of DSC and *F*_1_ scores and approaches the performance of Expert 2. On the no-new lesion cases, the proposed pipeline outperforms or achieves a comparable performance to Experts 1, 2 and 4.

#### 3.3.2. Sensitivity vs. specificity analysis

**Figure 4:**
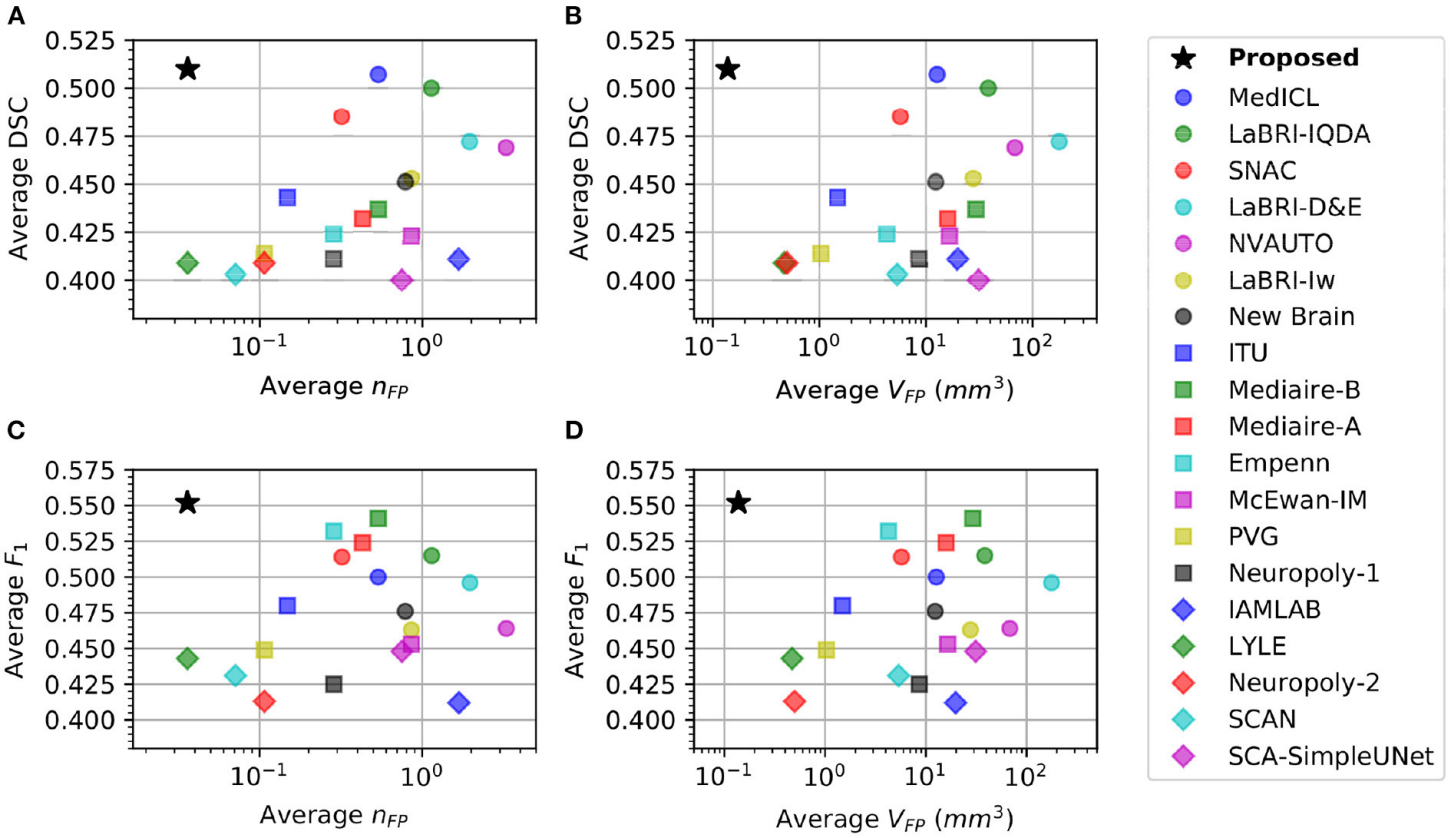
Comparison of different methods in new lesion metrics (DSC and *F*_1_) vs. no-new lesion metrics (*n*_*FP*_ and *V*_*FP*_). X-axis denotes one of the no-new lesion metrics in logarithmic scale and Y-axis denotes one of the new lesion metrics. Star denotes the proposed pipeline. **(A)** Plot of average DSC vs. average false positive lesion count *n*_*FP*_. **(B)** Plot of average DSC vs. average false positive lesion volume *V*_*FP*_. **(C)** Plot of average *F*_1_ vs. average false positive lesion count *n*_*FP*_. **(D)** Plot of average *F*_1_ vs. average false positive lesion volume *V*_*FP*_.

Table 1 shows that there is a reverse correlation between the results for new lesion cases vs. no-new lesions cases, especially in the participating methods for the MSSEG-2 challenge. In Figure 4, we plot the average DSC and *F*_1_ scores against the average *n*_*FP*_ and *V*_*FP*_ metrics. It shows that methods which perform well in new lesion cases do not perform as well in no-new lesion cases. Contrary to other methods, the proposed pipeline does not suffer from severe negative correlation, which performs well in both new lesion and no-new lesion cases.

#### 3.3.3. Comparison against state-of-the-art architectures

**Figure 5:**
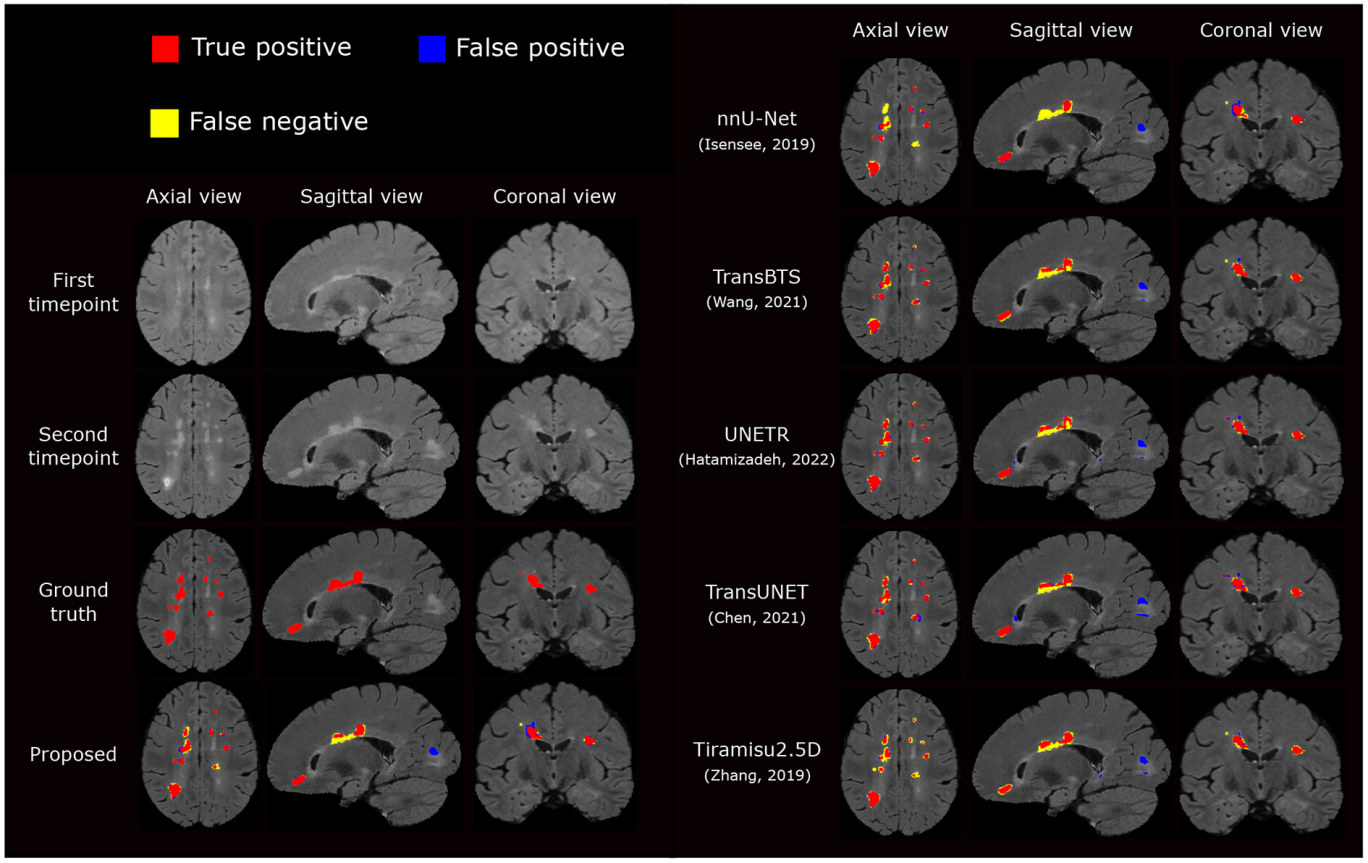
Visual comparison of the proposed segmentation pipeline to other methods. The proposed method produces a segmentation closest to the ground truth annotation.

We also compare the proposed pipeline to a number of state-of-the-art convolutional and transformer-based architectures, which have demonstrated excellent performance in biomedical image segmentation tasks. These architectures include the standard nnU-net (Isensee et al., 2021a), TransBTS (Wang et al., 2021), UNETR (Hatamizadeh et al., 2022), TransUNet (Chen et al., 2021), and Tiramisu 2.5D (Zhang et al., 2019). In order to evaluate methods fairly, we train these methods using the same preprocessed data, described in Section 2.2.1, which includes atlas-based brain extraction, N4 bias field correction, and free-form deformation registration, and use the standard data augmentation. The quantitative comparison results are reported in Table 2, and an example segmentation for visual comparison is provided in Figure 5. Table 2 shows that nnU-Net with standard data augmentations performs favorably against these state-of-the-art methods, and the proposed pipeline further improves performance possibly due to the additional data augmentation that we have introduced.

**Table 2:** Comparison of the proposed method to recent state-of-the-art deep learning architectures in terms of DSC and F1 scores, the number of false positive lesions *n*_*FP*_, the number of true positive lesions *n*_*TP*_, the number of false positive lesions *n*_*FP*_, the number of false negative lesions *n*_*FN*_, and volume of false positive lesions *V*_*FP*_ (unit: *mm*^3^), averaged across cases.

	**New lesion cases**					**No-new lesion cases**	
	**(*****n*** **= 32)**					**(*****n*** **= 28)**	
**Method**	**DSC**	***F_1_***	***n_TP_***	***n_FP_***	***n_FN_***	***n_FP_***	***V_FP_***
**Proposed**	**0.510**	**0.552**	4.969	2.031	2.281	**0.036**	0.192
nnU-Net	0.490	0.548	4.562	**1.281**	2.688	**0.036**	**0.138**
(Isensee et al., 2021a)							
TransBTS	0.477	0.470^*^	**5.492**	5.718^††^	**1.848**	0.939^†^	12.238
(Wang et al., 2021)							
UNETR	0.462	0.468^*^	5.343	9.031^†*††*^	1.906	4.214^†*††*^	23.705^*^
(Hatamizadeh et al., 2022)							
TransUNet	0.428^**^	0.434^**^	4.491	4.043^††^	2.102	1.081^††^	9.620
(Chen et al., 2021)							
Tiramisu 2.5D	0.363^***^	0.365^***^	4.313	4.625^††^	2.938	1.384^††^	15.120
(Zhang et al., 2019)							
Optimal score	1.000	1.000	7.000	0.000	0.000	0.000	0.000

The methods are sorted in the descending order of DSC. Best results are in bold. Asterisks indicate statistical significance (^*^p ≤ 0.05, ^**^p ≤ 0.01, and ^***^p ≤ 0.005) when using a paired Student's *t*-test compared to our proposed method. We implement the Mann-Whitney *U*-test for *n*_*TP*_, *n*_*FP*_, and *n*_*FN*_ metrics due to their non-normal distribution. The dagger symbols indicate statistical significance (^†^*p* ≤ 0.05, ^††^*p* ≤ 0.01, and ^†*††*^*p* ≤ 0.005) when using a Mann-Whitney *U*-test compared to the proposed method.

#### 3.3.4. Ablation study

We carry out an ablation study to evaluate the impacts of different components of the pipeline, including brain extraction and N4 bias correction (Pre), affine and free-form image registration (Reg) and additional data augmentation methods including axial subsampling and CarveMix (Aug+). By default, standard data augmentation methods are used which come with nnU-Net, described in Section 2.2.4. The ablation study results are presented in Table 3.

**Table 3:** Results for the ablation study, presenting DSC and *F*_1_ scores, the number of false positive lesions *n*_*FP*_, the number of true positive lesions *n*_*TP*_, the number of false positive lesions *n*_*FP*_, the number of false negative lesions *n*_*FN*_, and volume of false positive lesions *n*_*FP*_ (unit: *mm*^3^), averaged across cases.

			**New lesion cases**					**No-new lesion cases**	
			**(*****n*** **= 32)**					**(*****n*** **= 28)**	
**Pre**	**Reg**	**Aug+**	**DSC**	***F_1_***	***n_TP_***	***n_FP_***	***n_FN_***	***n_FP_***	***V_FP_***
			0.476	0.533	4.250	**1.281**	3.000	**0.000**	**0.000**
✓			0.475	0.524	4.688	**1.281**	2.563	**0.000**	**0.000**
	✓		0.473	0.525	4.188	1.343	3.062	0.036	0.083
✓	✓		0.490	0.548	4.562	**1.281**	2.688	0.036	0.138
✓	✓	✓	**0.510**	**0.552**	**4.969**	2.031	**2.281**	0.036	0.192

Best results are in bold.

Interestingly, adding pre-processing alone or registration alone does not drastically change performance metrics. However, when they are combined, for new lesion cases, the DSC score is increased from 0.476 to 0.490 and the *F*_1_ score is increased from 0.533 to 0.548. When imaging-related and lesion-aware data augmentations (Aug+) are introduced, the DSC score is further increased to 0.510 and the *F*_1_ score is increased to 0.552. This demonstrates that all the three components play an important role in the proposed pipeline. We also observe that when DSC and *F*_1_ scores are increased, metrics concerning no-new lesion cases become poorer. The undesired increase in false positive lesion count and lesion volume is discussed in detail in Section 3.3.6.

#### 3.3.5. Exclusion of no new lesion cases during training

The MSSEG-2 training dataset is composed of 40 subjects. 11 subjects do not exhibit new lesions in their follow-up scans. These subjects were removed from the training dataset, thus we only utilized 29 subjects. We carry out an additional study to investigate the impact of the exclusion of these images, by comparing the performance on the test set when utilizing all 40 subjects for segmentation model training against using the 29 subjects with new lesions. Results are presented in Table 4. Interestingly, removing the no new lesion subjects result in slightly higher DSC and *F*_1_ score, without compromising performance in the no new lesion cases. This is likely due to higher average representation of the foreground class (new lesions) in the altered training set. In addition, reducing the training set from 40 to 29 subjects decreased the model training time by 27.5%.

**Table 4:** Comparison between using the complete MSSEG-2 training set (40 subjects) against using 29 subjects which excludes the no new lesion cases.

	**New lesion cases**		**No-new lesion cases**	
	**(*****n*** **= 32)**		**(*****n*** **= 28)**	
**Training set**	**DSC**	***F_1_***	***n_FP_***	***V_FP_***
**29 subjects with new lesions**	**0.510**	**0.552**	**0.036**	**0.192**
All 40 subjects	0.502	0.530	**0.036**	**0.192**

We present the DSC and *F*_1_ scores, the number of false positive lesions *n*_*FP*_ and their volume *V*_*FP*_ (unit: *mm*^3^), averaged across cases. Best results are in bold.

#### 3.3.6. Sources of failure

**Figure 6:**
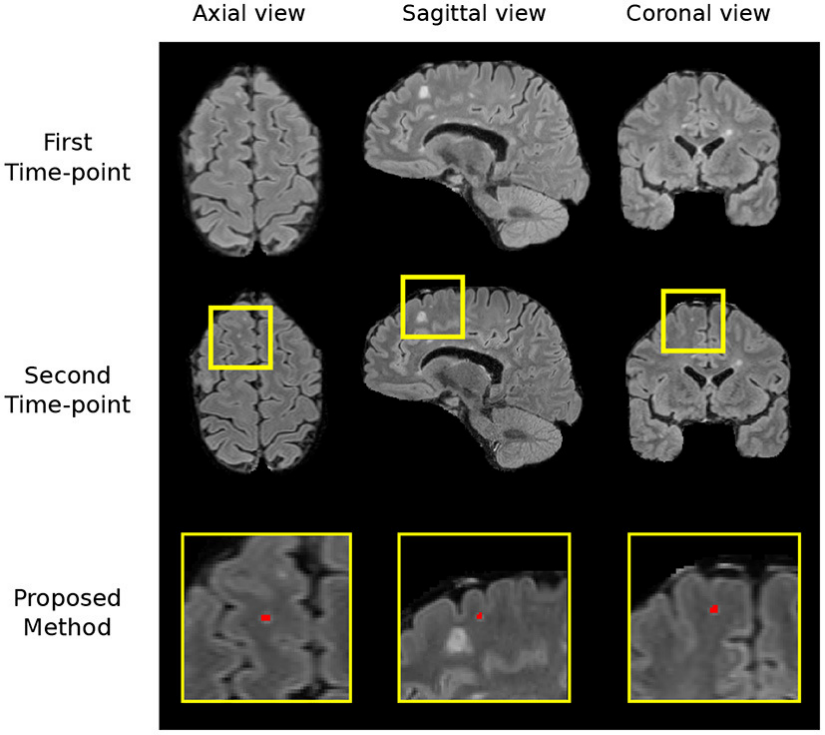
The region which we incorrectly classify as a lesion in the no-new lesion cases in the MSSEG-2 test set (subject ID: 004). We suspect the segmented region to be a new lesion, two of the human raters also classify this region as a new lesion.

We carry out a qualitative investigation on the test set to better understand where our method fails. In the no-new lesion cases, the proposed pipeline correctly classifies 27 out of the 28 subjects. The one misclassified (subject ID: 004) is incorrectly segmented to have 1 new lesion, which amount to 14 false positive voxels (3.875 mm^3^), shown in Figure 6. The segmented region has a higher intensity compared to surrounding regions and we suspect that it is likely to be a new lesion. Two of four expert raters also delineate this region as a new lesion, although the consensus segmentation does not regards this as a lesion, which leads to the misclassification of our method. This test set subject is the cause of the undesired increase in false positive lesion count and false positive lesion volume in Table 3.

**Figure 7:**
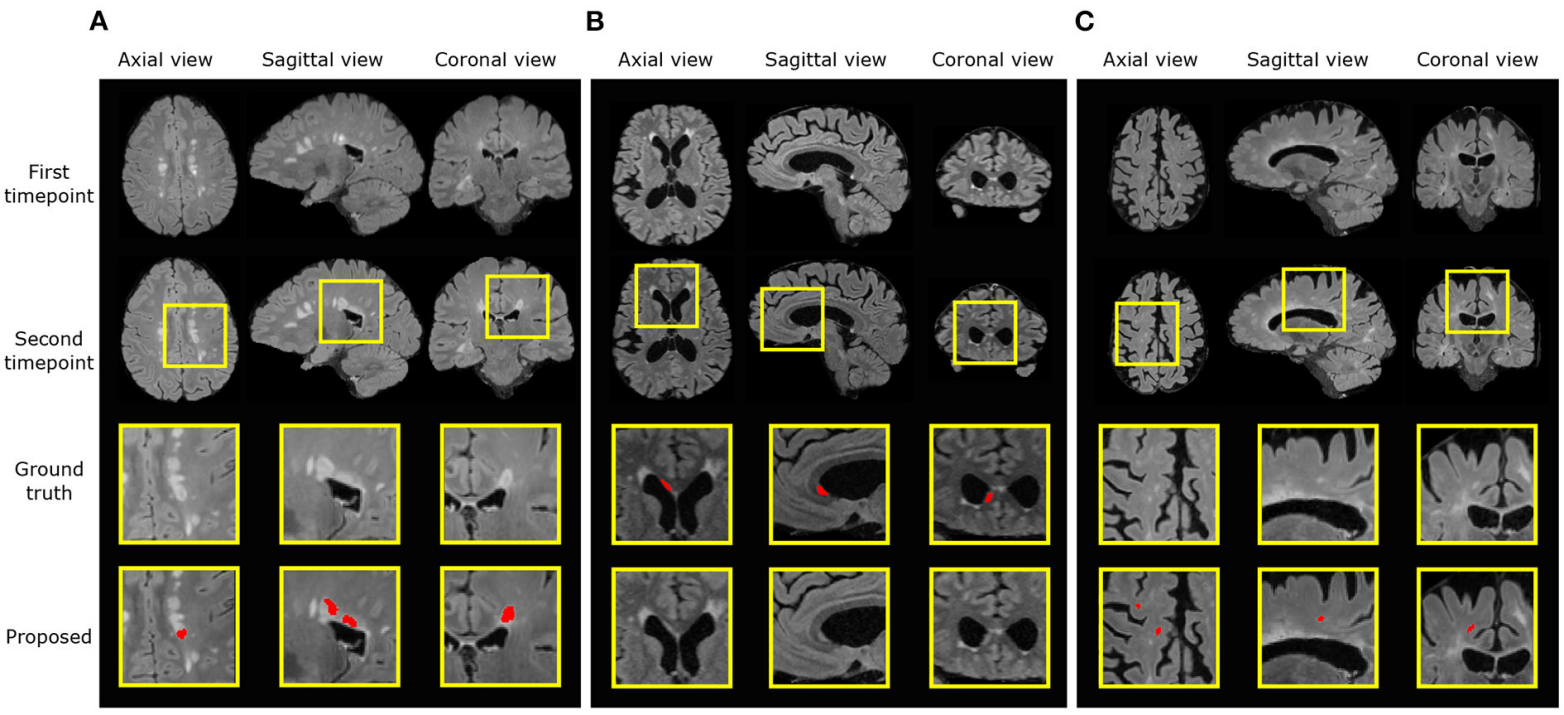
Examples of three different sources of error. Ground truth and predicted lesions are delineated in red. **(A)** (subject ID: 012) False positive segmentation of a growing lesion. **(B)** (subject ID: 078) False negative classification of a new lesion. **(C)** (subject ID: 036) False positive segmentation of a region classified as healthy/not-new in the consensus label, but annotated as a new lesion in two of the four provided expert annotations.

In the new lesion cases, when assessing against the DSC and *F*_1_ score, there is still room for improvement in performance. There are possibly three sources of failure that affect the DSC and *F*_1_ scores. The first is the incorrect segmentation of growing lesions. The pipeline employs affine and non-rigid registration to align the baseline scan to the follow-up and thus suppresses the detection of growing lesions. However, the remaining mis-alignment for some growing lesions still leads to the boundary voxels, i.e., the grown regions of lesions, being incorrectly segmented as new lesions. Secondly, the proposed pipeline may miss some tiny and less apparent new lesions. In some cases, new lesions which form in the follow-up scan are very small and less hyperintense compared to large new lesions. This makes the detection of these lesions very difficult and leads to misclassifications. Finally, new lesion segmentation is a generally challenging task even for human raters and there are indiscrepancies between annotations from different human experts. The noise in the annotations may limit what an automated method can achieve (Zhang et al., 2020). We present examples of all three sources of failure in Figure 7.

## 4. Discussion and conclusion

Here we demonstrate that by incorporating appropriate preprocessing steps, an nnU-net segmentation network, imaging and lesion-aware data augmentation techniques, we can achieve promising performance in new MS lesion segmentation tasks. The proposed pipeline outperforms other challenge participating methods in both new lesion cases and no-new lesion cases, in terms of DSC, *F*_1_, *n*_*FP*_ and *V*_*FP*_ scores. We also observe that in terms of network architecture, the recently popular transformer architectures may not necessarily outperform convolutional neural network architectures, such as nnU-net (Table 2). The design of proper pre-processing steps and problem-specific augmentations may play a more important role in this particular lesion segmentation task (Table 3).

In addition to the DSC and *F*_1_ score used by the MSSEG-2 challenge, we introduce extra evaluation metrics, *n*_*TP*_, *n*_*FP*_, and *n*_*FN*_, for the new lesion cases to understand the method performance. While many methods have a high *n*_*TP*_ and a lower *n*_*FN*_ score, the results suggest that a lower *n*_*FP*_ is what differentiates our method and the Experts to the other methods, thus providing a higher DSC and *F*_1_ score. The *n*_*TP*_, *n*_*FP*_, and *n*_*FN*_ results also suggest that they should be analyzed with respect to each other, as evaluating a method solely with one of these metrics can be misleading. For example, the top performing method in correctly identified average true positive lesions, *n*_*TP*_, ranks 10th in DSC score, and the top performing method in fewest average false positive lesion count ranks 17th in both DSC and *F*_1_ score. Methods with higher *n*_*TP*_ score also have high *n*_*FP*_ scores, with respect to Experts' performance. The results on the new metrics show that methods differ on their approach to achieve optimal DSC and *F*_1_ scores, and suggest that extra thought should be considered when evaluating a method solely on one metric.

Future efforts to improve the proposed method include further investigation of the sources of failure described in Section 3.3.6 and bridging the gap between automatic segmentation and expert raters. The current MSSEG-2 challenge dataset only contains annotations of new lesions. To discriminate new lesions from growing lesions, future works may include curating a dataset of both lesion types and training automated methods for detecting and differentiating these lesions. Also, additional post-processing steps could be developed to inspect local neighborhoods of detected new lesions and check whether they are connected to existing lesions or not, thus decreasing false positives for new lesion detection. However, too large of a local context may come at the cost of decreasing true positives too. Furthermore, the proposed pipeline only focuses on lesions in the brain region and the pre-processing step removes the spinal cord region. Despite the MSSEG-2 testing dataset not featuring any new lesions in the spinal cord, MS lesions can form in this region. Inclusion of the spinal cord into the preprocessing step and training data will extend the application of the proposed pipeline.

In conclusion, we propose an nnU-Net-based pipeline for multiple sclerosis new lesion segmentation. A contribution of the pipeline is that it incorporates task-specific data augmentation methods, including axial subsampling, which simulates MRI acquisition-based image artifacts, and CarveMix, which increases the diversity of lesion images. When evaluating on the MSSEG-2 dataset, the proposed pipeline achieves excellent performance in evaluation metrics for both new lesion and no-new lesion cases.

## Data availability statement

The original contributions presented in the study are included in the article/supplementary material, further inquiries can be directed to the corresponding authors.

## Author contributions

BB conducted all experiments and drafted the manuscript. WB provided significant assistance in technical issues and writing. PM provided guidance for the direction of research. All authors approved the final manuscript.

## Funding

This work is supported by the UKRI CDT in AI for Healthcare http://ai4health.io (Grant No. EP/S023283/1).

## Conflict of interest

The authors declare that the research was conducted in the absence of any commercial or financial relationships that could be construed as a potential conflict of interest.

## Publisher's note

All claims expressed in this article are solely those of the authors and do not necessarily represent those of their affiliated organizations, or those of the publisher, the editors and the reviewers. Any product that may be evaluated in this article, or claim that may be made by its manufacturer, is not guaranteed or endorsed by the publisher.

## Footnotes

1 Anima scripts: RRID:SCR_017072, https://anima.irisa.fr.

2 Anima scripts: RRID:SCR_017072, https://anima.irisa.fr.

3 Anima scripts: RRID:SCR_017072, https://anima.irisa.fr.
